# Associations of sitting accumulation patterns with cardio-metabolic risk biomarkers in Australian adults

**DOI:** 10.1371/journal.pone.0180119

**Published:** 2017-06-29

**Authors:** John Bellettiere, Elisabeth A. H. Winkler, Sebastien F. M. Chastin, Jacqueline Kerr, Neville Owen, David W. Dunstan, Genevieve N. Healy

**Affiliations:** 1 San Diego State University/University of California, San Diego | Joint Doctoral Program in Public Health (Epidemiology), San Diego, California, United States of America; 2 Center for Behavioral Epidemiology and Community Health, Graduate School of Public Health, San Diego State University, San Diego, California, United States of America; 3 The University of Queensland, School of Public Health, Brisbane, Queensland, Australia; 4 Institute for Applied Health Research, School of Health and Life Science, Glasgow Caledonian University, Glasgow, Scotland, United Kingdom; 5 Department of Movement and Sport Sciences, Faculty of Medicine and Health Sciences, Ghent University, Gent, East Flanders, Belgium; 6 Department of Family Medicine and Public Health, University of California San Diego, La Jolla, California, United States of America; 7 Baker IDI Heart and Diabetes Institute, Melbourne, Victoria, Australia; 8 Swinburne University of Technology, Melbourne, Victoria, Australia; 9 Mary McKillop Institute of Health Research, Australian Catholic University, Melbourne, Victoria, Australia; 10 School of Physiotherapy, Curtin University, Perth, Western Australia, Australia; Shanghai Diabetes Institute, CHINA

## Abstract

**Background:**

High amounts of time spent sitting can increase cardiovascular disease risk and are deleteriously associated cardio-metabolic risk biomarkers. Though evidence suggests that accruing sitting time in prolonged periods may convey additional risk, verification using high-quality measures is needed. We examined this issue in adults from the Australian Diabetes, Obesity and Lifestyle Study, using accurate measures of sitting accumulation.

**Methods:**

In 2011/12, 739 adults aged 36 to 89 years (mean±SD 58±10 years) wore activPAL3^™^ monitors (which provide accurate objective measures of sitting); 678 provided ≥4 valid days of monitor data and complete cardio-metabolic biomarker and confounder data. Multivariable linear regression models examined associations of sitting time, sitting time accrued in ≥30 minute bouts (prolonged sitting time), and three measures of sitting accumulation patterns with cardio-metabolic risk markers: body mass index (BMI), waist circumference, blood pressure, high- and low- density lipoprotein (HDL and LDL) cholesterol, triglycerides, glycated haemoglobin (HbA1c), fasting plasma glucose (FPG) and 2-hour post-load glucose (PLG). Interactions tests examined whether associations of sitting time with biomarkers varied by usual sitting bout duration.

**Results:**

Adjusted for potential confounders, greater amounts of sitting time and prolonged sitting time were significantly (p<0.05) deleteriously associated with BMI, waist circumference, HDL cholesterol, and triglycerides. Total sitting time was also significantly associated with higher PLG. Sitting accumulation patterns of frequently interrupted sitting (compared to patterns with relatively more prolonged sitting) were significantly beneficially associated with BMI, waist circumference, HDL cholesterol, triglycerides, PLG, and with FPG. Effect sizes were typically larger for accumulation patterns than for sitting time. Significant interactions (p<0.05) showed that associations of sitting time with HDL, triglycerides and PLG became more deleterious the longer at a time sitting was usually accumulated.

**Conclusions:**

Adding to previous evidence reliant on low-quality measures, our study showed that accumulating sitting in patterns where sitting was most frequently interrupted had significant beneficial associations with several cardio-metabolic biomarkers and that sitting for prolonged periods at a time may exacerbate some of the effects of sitting time. The findings support sedentary behavior guidelines that promote reducing and regularly interrupting sitting.

## Introduction

Diabetes mellitus and cardiovascular disease (CVD), on a global scale, account for more than one in four deaths annually [1]. In addition to lack of physical activity, sedentary behaviors—defined as time spent sitting or reclining while awake with low energy expenditure [2]—have emerged as a new risk factor [3–5]. Moreover, time spent sedentary has also been shown to be detrimentally associated with key biomarkers pertinent to both type 2 diabetes mellitus and CVD, notably excess adiposity and disordered lipid and glucose metabolism [6,7].

Australian and UK sedentary behavior guidelines [8,9] incorporate messages specifically targeting the reduction of prolonged sitting—that is, sitting for prolonged periods at a time. Reducing prolonged sitting time may yield benefits by reducing the total time spent sedentary and increasing activity, and may convey further benefits that are specific to reducing this type of sedentary behavior. Experimental studies have shown that by comparison with sitting that has been interrupted with small amounts of activity, sitting continuously for prolonged periods has acute detrimental effects on blood pressure and lipid metabolism [10–12] and on postprandial glucose control [13,14], with some effects persisting for up to 24 hours [10,15–18]. The observed beneficial effects could be attributed to breaking up sitting into shorter periods and/or to the small amounts of additional activity. Cross-sectional studies have observed statistically significant, detrimental associations of prolonged sitting time (variously defined) with waist circumference [19–21], BMI [19–21], HDL-cholesterol [20,21], triglycerides [20], and diastolic blood pressure [22]. Likewise, this may reflect benefits of sitting less and/or specifically avoiding sitting for long periods at a time. Contrary to results from experimental studies, cross-sectional studies have typically not observed significant associations between prolonged sitting and biomarkers of glucose control [19,21,22].

More rigorous evaluation of the effects of sedentary accumulation patterns is needed to better inform whether sedentary behavior guidelines [23] should be placing emphasis on prolonged sitting time and regularly interrupting sitting. Sedentary accumulation patterns refer to the degree to which sedentary time is accumulated in long, uninterrupted periods versus shorter, interrupted periods. Variously defined, sedentary accumulation patterns have shown cross-sectional associations with cardio-metabolic risk biomarkers, including BMI, waist circumference, insulin sensitivity, and triglycerides [6,17]. Many associations have persisted after statistical adjustment for the amount of time spent sedentary [7,20,22,24–26], suggesting that not all of the effects of prolonged sedentary accumulation patterns are produced by a greater amount of sedentary time they likely entail. When examined separately as time spent in long and short sedentary bouts, the effects of sedentary time have typically appeared larger for time spent in long bouts [19,21]. However, verification with valid measures is needed, as nearly all of the evidence regarding sedentary accumulation patterns has been derived using low-validity measures [27,28].

Using data from an activity monitor with good validity for measuring both sedentary behavior and sedentary accumulation patterns [27,29,30], we examined sedentary accumulation patterns in relation to cardio-metabolic biomarkers in a population-based study of Australian adults aged 35 years and over (n = 678). Specifically, we tested associations of sitting time, prolonged sitting time and sitting accumulation patterns with cardio-metabolic biomarkers. We also tested whether sitting time has associations with cardio-metabolic biomarkers that vary depending on how long at a time the sitting time was usually accumulated.

## Methods

### Sample and design

The Australian Diabetes, Obesity, and Lifestyle study (AusDiab) is a national, population-based cohort study established to understand the distribution and determinants of diabetes and other cardiovascular risk factors. Details of the original sampling methods and response rates are presented elsewhere [31]. Briefly, in 1999–2000, 11,247 adults aged ≥25 years completed questionnaires and underwent biomedical assessments. Participants were followed up in 2004–2005 [32] and again in 2011–2012 [33], with 4,562 adults (all now aged >35 years) attending one of the 46 testing centers across Australia in the 2011–2012 follow-up. Participants were ineligible for the 2011–2012 follow up if they requested not to be contacted, were deceased, moved overseas, or if they were severely/terminally ill and/or moved into a nursing facility classified for high care. A sub-sample of 1,014 participants attending the 2011–2012 onsite assessment were invited to join an ancillary study described in detail elsewhere [34] where participants were asked to wear activity monitors, including the activPAL3^™^, for seven consecutive days (beginning the next day). A total of 782 adults (77%) agreed to participate. Pregnant and/or non-ambulatory participants were not eligible for the ancillary study. All participants provided informed written consent. Protocols for the study were approved by the Alfred Health Human Ethics Committee (project no. 39/11).

### Measures

#### Cardio-metabolic outcomes

Upon arrival to the testing center, a fasting blood sample was drawn from each participant by venipuncture. Serum triglycerides, high-density lipoprotein (HDL) and total cholesterol were assayed by enzymatic methods. Low-density lipoprotein (LDL) cholesterol was estimated using the Freidewald equation [35]. Glycated hemoglobin (HbA1c) was measured by a high-performance liquid chromatography method (Bio-Rad Variance Hemoglobin Testing System; Bio-Rad, Hercules, CA, USA). Participants underwent a 75 g oral glucose tolerance test unless it was contraindicated (e.g., pregnancy, taking medication for diabetes). Fasting plasma glucose (FPG) and 2-hour post-load glucose (PLG) were determined by the hexokinase method using a Seimens Advia 2400 (Siemens AG, Munich, Germany). All blood specimens were analyzed at a central laboratory operated by Healhscope Pathology in Clayton, Victoria. Systolic and diastolic blood pressure were each calculated as the mean of the first two (of three) readings from an automated sphygmomanometer (Dinamap DP 101-NIBP; GE Medical Systems, Freiburg, Germany) after ≥ 5 minutes rest. Body mass index (BMI; kg/m^2^) was calculated from height and weight, measured to the nearest 0.5 cm and 0.1 kg (respectively) with participants removing shoes and excess clothing. Waist circumference was measured to the nearest 0.5 cm by tape measure between the lowest point on the ribs and the iliac crest on a horizontal plane, using the mean of two measures (or three measures, when the first two differed by ≥ 2 cm).

Minimum differences of interest (MDI) for the cardio-metabolic biomarkers, selected in discussion with a clinician to reflect clinically meaningful differences, were: 5% BMI (i.e., 1.36 kg/m^2^); 2 cm waist circumference; 5% HDL- and LDL-cholesterol (i.e., 0.08 and 0.15 mmol/L, respectively); 10% triglycerides (i.e., 0.11 mmol/L); 5 mmHg systolic blood pressure; 3 mmHg diastolic blood pressure; and 10% FPG, PLG and HbA1c (i.e., 0.54, 0.56, and 0.57 mmol/mol, respectively) [34].

#### Potential confounders

Socio-demographic, behavioral, and health-related characteristics measured by interviewer-administered questionnaire are described elsewhere [31] and listed in S1 Table. Overall energy intake (MJ/day), fiber intake (g/day), alcohol consumption (g/day), sodium intake (mg/day), and percentage of energy intake derived from fat and saturated fat were measured using the 80-item Dietary Questionnaire for Epidemiological Studies v2 [36].

#### Sedentary time and sedentary accumulation patterns

Being sedentary a certain number of times (bout frequency), each for a certain period (bout duration), adds up to the total volume of sedentary time [37] and, collectively, bout frequency and bout duration constitute sedentary accumulation patterns. There is no universally accepted indicator of sedentary accumulation. Most studies have examined “breaks” in sedentary time, which is a measure of how often people sit (when not accounting for sitting time) or of how often a certain amount of sedentary time is interrupted with activity (when accounting for the amount of sitting time). We examined three indicators of sedentary accumulation: transitioning from a seated to upright posture (sit-stand transitions, a similar concept to “breaks”), usual bout duration (also known as w50 or x50), and alpha [38]. Usual bout duration and alpha are theoretically sound measures of sedentary accumulation based on the distribution of sedentary bout duration [37,38] that each have slightly different measurement properties [37]. Usual bout duration is the midpoint of the cumulative distribution of sedentary bout durations (S1 Fig) [37,38]. Half of all sedentary time is accumulated in bouts longer than the usual bout duration. Alpha is a unitless measure that characterizes the frequency distribution of sedentary bout durations (S1 Fig) [38]. Lower values of alpha indicate sedentary time has been accumulated in relatively more long bouts and relatively fewer short bouts.

All of the activity measures were collected using the thigh-worn activPAL3^™^ monitor, which has high accuracy for measuring time spent sitting, standing, stepping and sitting accumulation patterns [27,29,39]. Rather than using the term sedentary, as this monitor specifically measures sitting, we refer to our results in terms of sitting time, prolonged sitting time (here, time spent sitting continuously for ≥30 minutes) and sitting accumulation patterns. The protocols and data processing procedures are described previously [34]. Briefly, participants were asked to wear the monitor 24 hours per day, and record sleep and monitor removals in a diary. Data were downloaded and initially processed using activPAL software version 6.4.1 (PAL Technologies Limited, Glasgow, UK) using default settings. Time spent sleeping, monitor non-wear, and invalid days (wear for <80% of waking hours and waking wear time <10 hours when diary data on sleep were missing) were removed using the diary and monitor data. Totals each day, averaged across valid days, were obtained for the number of sit-stand transitions and time spent: sitting; sitting in ≥30 min bouts; standing; stepping; and, stepping at ≥ 3 METs (i.e., moderate to vigorous physical activity; MVPA). The residuals method [40,41] was used to correct sitting time, prolonged sitting time, and MVPA for waking wear time, and to correct sit-stand transitions for sitting time. The accumulation measures were calculated as outlined elsewhere [38,42] (and detailed in S1 Fig).

### Statistical analyses

Out of the initial monitor subsample (n = 782), only participants who wore the monitor (n = 741) for at least four valid days (n = 720) who were not pregnant (n = 718) and provided data on covariates and outcomes (n = 678, and n = 639 for PLG) were included. All statistical analyses were performed using Stata 14.0 (StataCorp LP, College Station, TX, USA) using linearized variance estimation to account for the survey design of AusDiab3. Significance was set at p<0.05 (two-tailed).

Multivariable linear regression was used to model the associations with each cardio-metabolic outcome of the prolonged sitting time and sitting accumulation patterns, adjusting for age, gender, and potential confounders. Results for sitting time have been reported previously [34] and are included here to place the effect sizes observed for prolonged sitting time and sitting accumulation patterns in context. Log transformation was used to improve the normality of BMI, triglycerides, HbA1c, glucose, and PLG. The sitting-related exposures were all examined as quintiles, with the first quintile (Q1; the referent category) always denoting the most time spent sitting or most prolonged (i.e., least interrupted) sitting accumulation pattern (see S2 Table). From the linear regression models, we report on pairwise comparisons of marginal means with all covariates set to their mean values, overall p-values, and p-for-trend. Potential confounders (S1 Table) were determined for each outcome using backwards elimination (p<0.20 for retention). Detrimental effects on biomarkers may occur through sitting displacing MVPA and via increases in body weight. Though MVPA and BMI could be confounders and/or causal intermediates, they were treated as the latter and therefore not adjusted as potential confounders in the main analyses [43]. MVPA-adjusted results are in S5 and S6 Tables to allow comparison to results of prior research and assess how sensitive conclusions were to the choice to adjust or not adjust. Age and gender interactions were explored in all models with a strict level of significance of p<0.001 because of the large number of tests performed.

Models do not adjust for sitting time as a confounder [44] because increasing the volume of sitting is one of the ways in which sitting for long periods may impact biomarkers. Instead, we tested whether the associations with the biomarker outcomes of sitting time (h/day, mean-centered) varied by usual bout duration (minutes, mean-centered) using interaction terms. Interactions meeting a generous threshold of p<0.1 were reported. To better describe the magnitude of any interaction detected, we report what the effects each hour per day of sitting time were when accumulated in “very long” and “very short” bouts. The mean value of Q1 and Q5 were used to represent “very long” and “very short” bouts.

## Results

The analytic sample included 678 adults (n = 639 for analyses of PLG) with a mean age (± SD) of 57.8±9.8 years, after excluding participants who were pregnant or had any missing data (Table 1). Additional participant characteristics are described in Table 1 and S3 Table.

**Table 1:** Selected sociodemographic, medical, cardio-metabolic, and sitting-related characteristics of the final analytic sample, (AusDiab 2011–12; n = 678).

Age, years	57.8 (9.8)
Men, n(%)	297 (45)
Ethnicity, n(%)	
Australia/New Zealand (Non-Indigenous)	550 (81)
Australia/New Zealand (Aboriginal/Torres Strait Islander)	4 (1)
Other English speaking	75 (11)
South Europe	8 (1)
Other Europe	19 (3)
Asia	18 (3)
Other	4 (1)
Family history of diabetes, n(%)	191 (28)
Prior cardiovascular disease diagnosis^a^, n(%)	41 (6)
Body Mass Index, kg/m2	27.4 (4.9)
Waist circumference, cm	93 (13.7)
Systolic blood pressure, mmHg	126.3 (17.3)
Diastolic blood pressure, mmHg	72.7 (10.5)
Fasting blood glucose, mmol/L	5.3 (0.73)
HbA1c (IFCC), mmol/L	5.6 (0.35)
High-density lipoprotein cholesterol, mmol/L	1.6 (0.41)
Low-density lipoprotein cholesterol, mmol/L	3.0 (0.82)
Triglycerides, mmol/L	1.3 (0.66)
2-hour postload plasma glucose, mmol/L^b^	5.6 (2.02)
Daily sitting time^c^^,^^f^, h/day	8.8 (1.7)
Time in sedentary bouts ≥30 minutes ^c^^,^^f^, h/day	4.0 (1.6)
Sit-stand transitions^d^, n/day	54.1 (14.5)
Usual bout duration (min)	26.2 (8.9)
Alpha^g^	1.3 (0.039)
Moderate to Vigorous Physical Activity^e^^,^^f^, h/day	1.2 (0.4)

Abbreviations: IFCC, International Federation of Clinical Chemistry and Laboratory Medicine

Table reports mean (standard deviation) or n(%); means, standard deviations, and % are corrected for the complex sampling design using linearized variance estimation. Results are for the analytic sample that was obtained using complete case analysis.

^a^ Heart attack, stroke or angina.

^b^ 2-hr postload plasma glucose data were missing from 39 participants.

^c^ Variables standardized to device wear time using the residuals method.

^d^ Variable standardized to daily sitting time using the residuals method.

^e^ Measured via activPAL as "stepping" equivalent to ≥ 3 METs.

^f^ Estimates are similar to those previously reported in Healy et al. Eur Heart J. 2015, differing slightly due to small differences in inclusion criteria.

^g^ Alpha is a unitless measure of sitting accumulation ranging from 1.22 to 1.51. Higher values indicate accumulation patterns with relatively more interrupted sitting than prolonged sitting.

**Fig 1:**
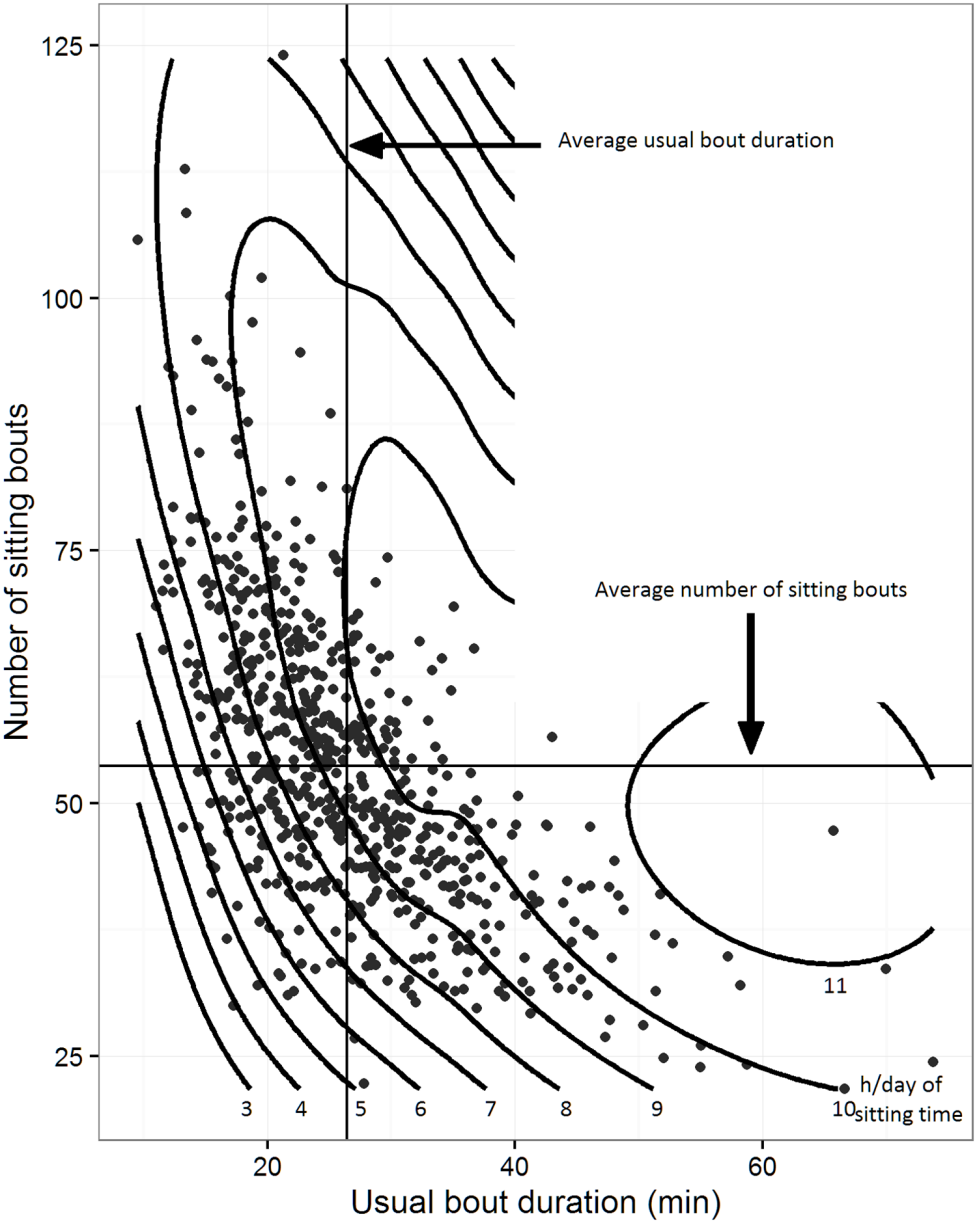
Sitting accumulation patterns. Number of bouts (y axis), usual bout duration (x axis) and the amount of sitting time accumulated (contour lines). Each point represents one participant.

Fig 1 depicts participants’ sitting accumulation patterns (bout frequency and bout duration) in relation to total sitting volume. The longer at a time that participants usually accumulated their sitting, typically the fewer the number of sitting bouts. An increase in either bout frequency or bout duration appeared to be non-linearly related to accruing a greater amount of sitting time. Sitting times of 6 to <10 h/day were seen over a diverse range and combination of sitting bout frequencies and durations. Notably, sitting times of 10 h/day or more almost exclusively occurred with above-average usual bout duration. Sitting times of < 6 h/day almost exclusively occurred when participants had both fewer and shorter bouts than average.

### Associations with cardio-metabolic biomarkers

Table 2 shows the associations of daily sitting time, prolonged sitting time, and sitting accumulation patterns with measures of adiposity and lipid measures. BMI and waist circumference decreased significantly across increasing quintiles of each of the measures. Mean differences (95% CI) between the top and bottom quintiles (Q5 versus Q1) were often of a sizeable magnitude (i.e., equivalent to the MDI or greater), ranging from -1.34 (-2.55, -0.13) to -3.54 (-4.90, -2.18) kg/m^2^ for BMI and -3.48 (-6.69, -0.27) to -10.54 (-13.93, -7.16) cm for waist circumference. The observed differences were largest by alpha and smallest by sitting time. Only sitting time, prolonged sitting time, and alpha showed significant associations with HDL-cholesterol and triglycerides. These observed differences were also sizeable, ranging 0.14–0.15 mmol/L (HDL-cholesterol) and 0.20–0.29 mmol/L triglycerides. Associations of the other sitting accumulation measures with HDL-cholesterol and triglycerides were also beneficial in direction, but weaker and non-significant. No significant associations were observed with LDL cholesterol.

**Table 2:** Associations and all potential covariates, of sitting and prolonged sitting time, and sitting accumulation with measures of adiposity and lipid metabolism in Australian adults aged 36 to 80 (AusDiab 2011–12; n = 678).

	Quintile 1^a^	Quintile 2		Quintile 3		Quintile 4		Quintile 5		P-for-trend	P-overall
		Mean diff.^b^	95% CI	Mean diff.^b^	95% CI	Mean diff.^b^	95% CI	Mean diff.^b^	95% CI		
**Body Mass index (kg/m2)**											
Total sitting time^c^^,^^d^	referent	0.28	(-0.91,1.48)	-0.70	(-1.55,0.25)	-1.57	(-2.45,-0.69)	-1.34	(-2.55,-0.13)	**0.001**	**<.001**
Prolonged sitting time^c^	referent	0.29	(-0.93,1.51)	-0.90	(-2.00,0.25)	-1.40	(-2.47,-0.33)	-1.64	(-2.82,-0.46)	**0.001**	**0.011**
Sit-stand transitions^e^	referent	-0.57	(-1.53,0.39)	-1.50	(-2.56,-0.48)	-1.26	(-2.44,-0.08)	-2.76	(-3.82,-1.70)	**<.001**	**<.001**
Usual bout duration	referent	-1.26	(-2.32,-0.21)	-1.20	(-2.27,-0.11)	-1.32	(-2.54,-0.10)	-2.04	(-3.34,-0.74)	**0.007**	0.058
Alpha	referent	-0.30	(-1.53,0.92)	-1.70	(-3.11,-0.20)	-2.33	(-3.65,-1.02)	-3.54	(-4.90,-2.18)	**<.001**	**<.001**
**Waist circumference (cm)**											
Total sitting time^c^^,^^d^	referent	-0.49	(-3.47,2.49)	-2.10	(-4.76,0.48)	-5.46	(-7.74,-3.17)	-3.48	(-6.69,-0.27)	**0.001**	**<.001**
Prolonged sitting time^c^	referent	0.16	(-3.15,3.46)	-3.00	(-6.11,0.01)	-4.12	(-6.73,-1.51)	-4.22	(-7.25,-1.20)	**0.001**	**0.012**
Sit-stand transitions^e^	referent	-2.39	(-4.80,0.03)	-4.80	(-7.52,-2.06)	-3.70	(-6.43,-0.97)	-7.46	(-10.32,-4.59)	**<.001**	**<.001**
Usual bout duration	referent	-2.90	(-6.01,0.22)	-3.20	(-6.14,-0.18)	-3.90	(-6.82,-0.98)	-4.97	(-8.11,-1.83)	**0.004**	0.057
Alpha	referent	-2.30	(-5.26,0.67)	-5.60	(-9.1,-2.04)	-7.29	(-10.55,-4.02)	-10.54	(-13.93,-7.16)	**<.001**	**<.001**
**HDL Cholesterol (mmol/L)**											
Total sitting time^c^^,^^d^	referent	0.01	(-0.06,0.09)	0.10	(-0.02,0.14)	0.16	(0.08,0.25)	0.14	(0.07,0.22)	**<.001**	**<.001**
Prolonged sitting time^c^	referent	0.09	(0.00,0.17)	0.10	(-0.02,0.17)	0.12	(0.06,0.18)	0.14	(0.06,0.22)	**<.001**	**0.001**
Sit-stand transitions^e^	referent	0.03	(-0.07,0.13)	0.00	(-0.07,0.10)	0.03	(-0.06,0.12)	0.06	(-0.04,0.15)	0.266	0.673
Usual bout duration	referent	0.05	(-0.02,0.11)	0.00	(-0.05,0.08)	0.02	(-0.04,0.08)	0.06	(0.00,0.13)	0.129	0.356
Alpha	referent	0.03	(-0.05,0.12)	0.10	(0.02,0.20)	0.09	(0.02,0.16)	0.15	(0.07,0.24)	**<.001**	**0.002**
**LDL Cholesterol (mmol/L)**											
Total sitting time^c^^,^^d^	referent	0.07	(-0.06,0.21)	0.00	(-0.19,0.13)	-0.02	(-0.17,0.14)	0.00	(-0.17,0.18)	0.676	0.807
Prolonged sitting time^c^	referent	0.08	(-0.08,0.24)	0.00	(-0.14,0.23)	0.06	(-0.14,0.26)	0.05	(-0.12,0.23)	0.669	0.907
Sit-stand transitions^e^	referent	0.05	(-0.16,0.27)	0.00	(-0.22,0.16)	0.00	(-0.22,0.22)	0.10	(-0.09,0.29)	0.436	0.774
Usual bout duration	referent	-0.03	(-0.24,0.18)	0.10	(-0.09,0.23)	-0.03	(-0.24,0.17)	0.12	(-0.06,0.29)	0.247	0.370
Alpha	referent	0.03	(-0.15,0.21)	0.00	(-0.19,0.19)	0.00	(-0.17,0.16)	-0.01	(-0.16,0.14)	0.756	0.993
**Triglycerides (mmol/L)**											
Total sitting time^c^^,^^d^	referent	-0.04	(-0.17,0.08)	-0.10	(-0.25,-0.03)	-0.32	(-0.41,-0.22)	-0.23	(-0.35,-0.11)	**<.001**	**<.001**
Prolonged sitting time^c^	referent	-0.13	(-0.26,0.00)	-0.20	(-0.34,-0.07)	-0.18	(-0.29,-0.07)	-0.20	(-0.32,-0.08)	**0.003**	**0.007**
Sit-stand transitions^e^	referent	0.02	(-0.10,0.15)	0.00	(-0.17,0.12)	-0.07	(-0.20,0.06)	-0.09	(-0.24,0.05)	0.093	0.406
Usual bout duration	referent	-0.09	(-0.23,0.05)	-0.10	(-0.22,0.02)	-0.05	(-0.18,0.07)	-0.11	(-0.24,0.03)	0.286	0.210
Alpha	referent	-0.13	(-0.25,0.00)	-0.10	(-0.25,0.03)	-0.16	(-0.29,-0.03)	-0.29	(-0.41,-0.18)	**<.001**	**<.001**

^a^ Participants in quintile 1 have the highest total sitting time / prolonged sitting time / the most prolonged pattern of sitting time accumulation. Quintile cutpoints are in S2 Table.

^b^ Difference in adjusted mean in contrast to quintile 1, adjusted for age, gender and potential confounders (S1 Table), from linear regression model with linearized variance estimation accounting for state/testing center strata/clusters.

^c^ Variables adjusted for device wear time using the residuals method.

^d^ Associations are similar to those previously reported in Healy et al. Eur Heart J. 2015, differing slightly due to small differences in inclusion criteria and differences in the functional form of total sitting time.

^e^ Variable adjusted for total sitting time using the residuals method.

Bolded p-values indicate statistically significant relations at p < 0.05.

Table 3 shows the results for blood pressure and glucose. All associations with blood pressure and HbA1c were small (i.e., less than the MDI) and not statistically significant. Only alpha showed a statistically significant association with FPG; a small difference favoring patterns with more interrupted sitting (-0.20, 95% CI: -0.36, -0.04 mmol/L for Q5 versus Q1) was observed. Significantly lower PLG was observed with less sitting time (-0.50, 95% CI: -0.85, -0.14 mmol/L for Q5 versus Q1) and higher alpha (-0.64, 95% CI: -1.00, -0.29 mmol/L for Q5 versus Q1).

**Table 3:** Associations of sitting and prolonged sitting time, and sitting accumulation with measures of blood pressure and glucose control in Australian adults aged 36 to 80 (AusDiab 2011–12; n = 678^a^).

	Quintile 1^b^	Quintile 2		Quintile 3		Quintile 4		Quintile 5		P-for-trend	P-overall
		Mean diff.^c^	95% CI	Mean diff.^c^	95% CI	Mean diff.^c^	95% CI	Mean diff.^c^	95% CI		
**Systolic BP (mmHg)**											
Total sitting time^d^^,^^e^	referent	0.69	(-2.44,3.82)	-1.60	(-5.10,1.84)	1.43	(-2.46,5.31)	1.05	(-1.83,3.93)	0.443	0.572
Prolonged sitting time^d^	referent	2.68	(-2.02,7.37)	4.70	(-0.18,9.49)	2.17	(-2.08,6.41)	2.84	(-1.27,6.95)	0.243	0.379
Sit-stand transitions^f^	referent	-2.14	(-5.66,1.38)	-1.80	(-5.78,2.14)	-1.40	(-5.86,3.05)	-0.50	(-5.05,4.06)	0.978	0.724
Usual bout duration	referent	0.12	(-4.20,4.43)	3.30	(-0.67,7.33)	0.88	(-3.87,5.63)	1.84	(-2.06,5.73)	0.366	0.562
Alpha	referent	1.36	(-2.51,5.23)	0.40	(-4.34,5.10)	-0.07	(-5.47,5.33)	0.52	(-4.00,5.05)	0.937	0.944
**Diastolic BP (mmHg)**											
Total sitting time^d^^,^^e^	referent	1.94	(-0.17,4.04)	-1.40	(-3.38,0.67)	0.01	(-2.25,2.27)	-0.98	(-3.12,1.17)	0.158	0.058
Prolonged sitting time^d^	referent	2.52	(0.37,4.66)	1.90	(-0.89,4.75)	0.81	(-1.65,3.28)	1.20	(-1.39,3.80)	0.845	0.290
Sit-stand transitions^f^	referent	0.59	(-1.60,2.78)	-0.60	(-3.03,1.81)	-0.67	(-3.21,1.87)	-0.18	(-2.92,2.57)	0.613	0.810
Usual bout duration	referent	0.44	(-1.66,2.53)	0.90	(-1.64,3.34)	0.45	(-2.13,3.04)	1.05	(-1.52,3.62)	0.489	0.946
Alpha	referent	-0.47	(-2.56,1.62)	-1.60	(-4.14,1.00)	-1.96	(-4.71,0.80)	-2.05	(-4.54,0.44)	0.082	0.486
**HbA1c (mmol/mol)**											
Total sitting time^d^^,^^e^	referent	0.05	(-0.06,0.15)	0.00	(-0.09,0.08)	-0.05	(-0.14,0.05)	0.00	(-0.09,0.08)	0.332	0.481
Prolonged sitting time^d^	referent	0.04	(-0.06,0.14)	0.00	(-0.10,0.12)	0.00	(-0.07,0.08)	0.01	(-0.07,0.09)	0.813	0.915
Sit-stand transitions^f^	referent	-0.03	(-0.12,0.06)	0.00	(-0.09,0.17)	0.03	(-0.05,0.11)	0.01	(-0.09,0.11)	0.428	0.563
Usual bout duration	referent	0.06	(-0.02,0.15)	0.10	(-0.03,0.17)	0.06	(-0.03,0.15)	0.04	(-0.03,0.11)	0.328	0.530
Alpha	referent	0.01	(-0.09,0.12)	0.00	(-0.14,0.08)	-0.03	(-0.12,0.07)	-0.04	(-0.12,0.05)	0.246	0.767
**Fasting glucose (mmol/L)**											
Total sitting time^d^^,^^e^	referent	0.12	(-0.07,0.30)	0.00	(-0.2,0.16)	-0.10	(-0.29,0.09)	-0.13	(-0.35,0.10)	0.070	0.135
Prolonged sitting time^d^	referent	0.04	(-0.13,0.21)	0.10	(-0.12,0.30)	-0.03	(-0.18,0.11)	-0.08	(-0.33,0.17)	0.353	0.436
Sit-stand transitions^f^	referent	-0.08	(-0.27,0.11)	0.00	(-0.20,0.25)	0.05	(-0.17,0.27)	0.03	(-0.15,0.21)	0.361	0.454
Usual bout duration	referent	0.05	(-0.13,0.23)	0.10	(-0.14,0.30)	0.11	(-0.09,0.32)	-0.04	(-0.25,0.17)	0.907	0.476
Alpha	referent	-0.05	(-0.28,0.18)	-0.30	(-0.45,-0.12)	-0.15	(-0.31,0.02)	-0.20	(-0.36,-0.04)	**<.001**	**<.001**
**2-hour post-load glucose (mmol/L)**											
Total sitting time^d^^,^^e^	referent	-0.29	(-0.77,0.20)	-0.40	(-0.82,0.09)	-0.36	(-0.78,0.07)	-0.50	(-0.85,-0.14)	**0.009**	0.116
Prolonged sitting time^d^	referent	-0.21	(-0.52,0.11)	-0.40	(-0.74,0.04)	-0.33	(-0.71,0.04)	-0.18	(-0.60,0.24)	0.326	0.438
Sit-stand transitions^f^	referent	-0.16	(-0.57,0.25)	-0.10	(-0.38,0.25)	-0.34	(-0.77,0.08)	-0.12	(-0.57,0.32)	0.422	0.502
Usual bout duration	referent	-0.16	(-0.42,0.11)	-0.20	(-0.67,0.18)	-0.16	(-0.51,0.19)	0.02	(-0.37,0.40)	0.930	0.474
Alpha	referent	-0.40	(-0.80,0.00)	-0.20	(-0.55,0.10)	-0.71	(-1.20,-0.21)	-0.64	(-1.00,-0.29)	**0.002**	**0.009**

^a^ Models of 2-hour post-load glucose had n = 639.

^b^ Participants in quintile 1 have the highest total sitting time / prolonged sitting time / the most prolonged pattern of sitting time accumulation. Quintile cutpoints are in S2 Table.

^c^ Difference in adjusted mean in contrast to quintile 1, adjusted for age, gender and potential confounders (S1 Table), from linear regression models with linearized variance estimation accounting for state/testing center strata/clusters.

^d^ Variables adjusted for device wear time using the residuals method.

^e^ Associations are similar to those previously reported in Healy et al. Eur Heart J. 2015, differing slightly due to small differences in inclusion criteria and differences in the functional form of total sitting time.

^f^ Variable adjusted for total sitting time using the residuals method.

Bolded p-values indicate statistically significant relations at p < 0.05.

None of the associations reported in Tables 2 and 3 differed by age or gender at p<0.001 (S4 Table). Mostly, the associations reported in Tables 2 and 3 were only partially attenuated by statistical adjustment for MVPA (S5 and S6 Tables). Complete loss of significance was observed only for associations of usual bout duration with adiposity, and of sitting time with PLG.

### Effect modification by usual bout duration

**Fig 2:**
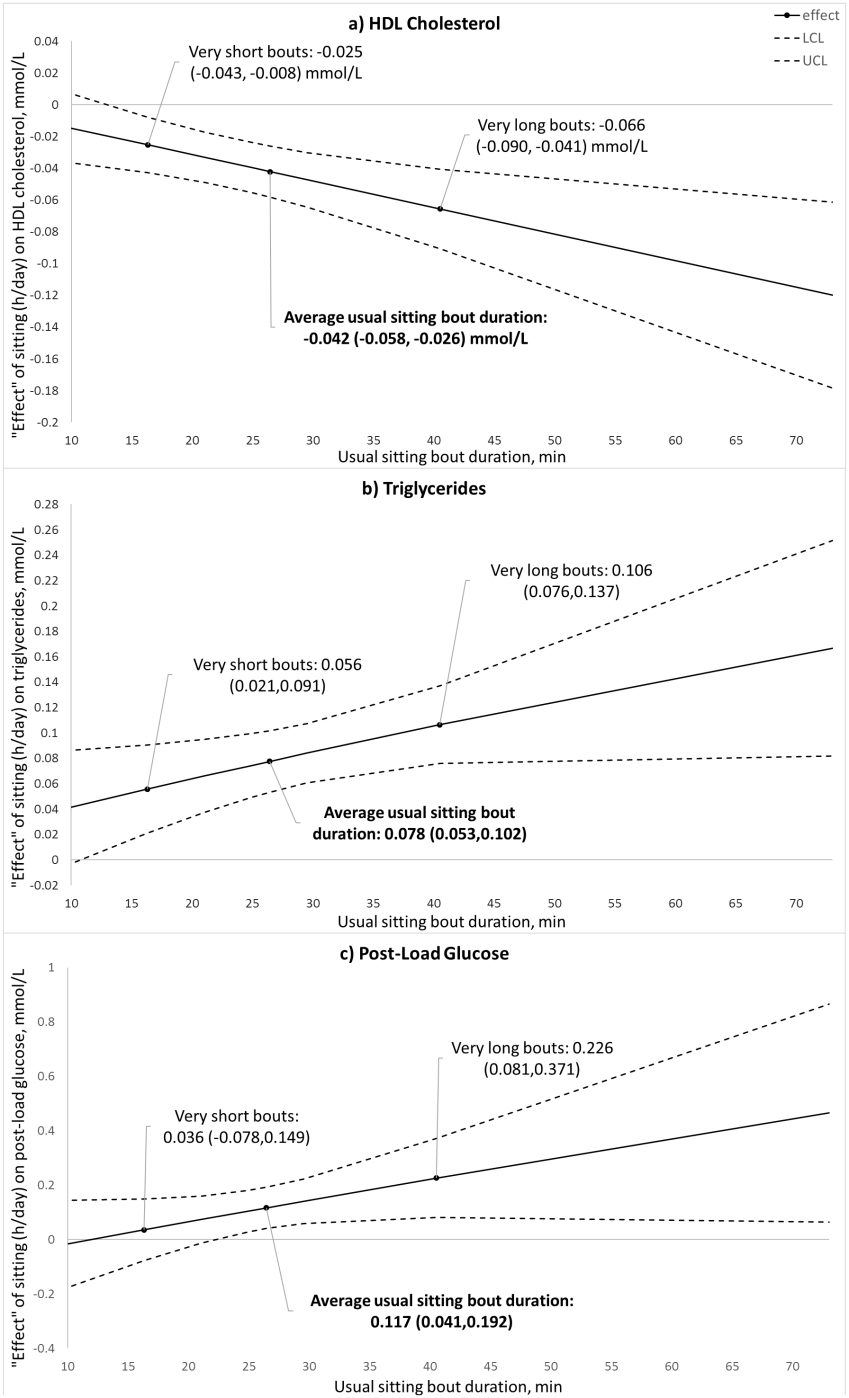
Cross-sectional associations of each hour of daily sitting time with (a) HDL-cholesterol, (b) triglycerides, and (c) PLG glucose by how long at a time the sitting was usually accumulated. Solid lines indicate the estimated association between total sitting time and each biomarker with the dashed lines indicating 95% confidence intervals.

Usual sitting bout duration significantly modified associations of sitting time with HDL-cholesterol (p = 0.005), triglycerides (p = 0.032), and PLG (p = 0.042) (S7 Table). In all instances, sitting time showed associations with biomarkers that were more strongly detrimental the longer at a time that sitting time was usually accumulated (Fig 2). For example, at an average usual bout duration (26.2 minutes), each hour per day spent sitting was associated with 0.04 (95% CI: 0.03, 0.06) mmol/L lower HDL cholesterol (Fig 2). By contrast, this lowering of HDL cholesterol with each hour per day spent sitting was 0.03 (95% CI: 0.01, 0.04) mmol/L with sitting time usually accumulated in very short bouts and 0.07 (95% CI: 0.04, 0.09) mmol/L with sitting time usually accumulated in very long bouts (Fig 2).

## Discussion

This study evaluated sedentary accumulation in relation to cardio-metabolic biomarkers in a large, general population sample of adults. To our knowledge, this study is among the first to examine this topic with accurate measures of sitting accumulation [37,38]. Many of the previous findings concerning sedentary accumulation that had been established with low-validity measures were corroborated. Although sitting many times per day and accumulating sitting time in long bouts were both relevant in terms of how much sitting time adults ultimately accrued, additional time spent sitting did not appear to be the only relevant correlate of prolonged accumulation patterns.

The only other study of which the authors are aware that has tested associations of patterns with adult cardiometabolic biomarker outcomes using high-quality measures of sitting patterns was the Maastricht Study [45]. Adjusting only for confounders, not competing time uses, the authors found that sitting patterns (measured as breaks, number of ≥30 min sitting bouts and average sitting bout duration) had statistically significant associations with both metabolic syndrome and glucose metabolism status (normal, impaired fasting glucose/impaired glucose tolerance, type 2 diabetes mellitus). Effects typically indicated the healthiest participants had the most interrupted sedentary patterns though associations were not significant with all of the pattern measures. A meta-analysis of associations between adiposity and sedentary accumulation patterns concluded that there is “some certainty” that more interrupted patterns (specifically, more breaks in sedentary time) are significantly associated with lower BMI and, with less certainty, smaller waist circumference [17]. These same associations were present using our three indicators of sitting accumulation patterns. Previously, a review had concluded there was “some evidence” that sedentary accumulation patterns are associated with triglycerides and there was “inconclusive evidence” of an association with HDL-cholesterol [6]. Our findings were in favor of an association with both cardio-metabolic biomarkers. Consistent with prior studies [21,22], we did not observe significant associations of sitting accumulation patterns with LDL cholesterol. The null results for HbA1c were consistent with the typically null results in the extant literature [19,21] and our null results for blood pressure did not conflict with prior results, which are mixed [19,22,24,46]. Despite the potential biases in previous findings, our findings with high-quality measures did not contradict any of the previous conclusions regarding these biomarkers.

The greatest dissimilarity between our findings and the extant literature based on observational studies was for glucose metabolism. In most—but not all [22,24]—previous cross-sectional research, significant associations of sedentary accumulation patterns with FPG and PLG have not been observed [6]. Notably, our findings were dependent on the accumulation measure employed; both these associations were detected only with alpha. Similarly, in the Maastricht study, associations with glucose metabolism were not significant for “breaks” as a measure but were significant for average bout duration and number of prolonged bouts [45]. It is possible that different indicators of accumulation patterns may have different capabilities to detect true effects, and different susceptibilities to unmeasured confounders. Alternatively, our findings could be an aberration or the result of multiple hypothesis testing.

Though the adjustment or non-adjustment for MVPA is a contentious issue on both epidemiologic and statistical grounds [43,47], it did not appear to strongly affect what conclusions were drawn in our study concerning sedentary accumulation patterns. Adjustment for MVPA (not a procedure we advocate in general) led to only partial attenuation of results—seldom to complete loss of statistical significance. The limited degree of attenuation also suggests that the beneficial associations with cardio-metabolic biomarkers that we observed for our sitting-related exposures are likely to involve mechanisms other than those induced by, or operating through, additional MVPA. By contrast, causation more generally, particularly as concerns adiposity, remains unresolved and is important to establish in further research with longitudinal and/or experimental designs. Many of our findings could be explained by heavier bodyweight inducing individuals to transition between postures less frequently.

The present study provides some evidence to support prolonged sitting as a specific target of sedentary reduction messages. Waist circumference, BMI, HDL cholesterol and triglycerides were significantly associated with sitting time and prolonged sitting time. PLG was further significantly associated with total sitting time. Although effect sizes for these outcomes were similar when examining sitting and prolonged sitting, rather than suggesting all types of sitting are the same, this likely reflects the problems in examining only one subtype of sitting in isolation. Comparing participants according to their sitting accumulation patterns consistently showed greater differences between the top and bottom quintiles than either sitting or prolonged sitting time, especially by the alpha measure. Crucially, the longer at a time participants accumulated their sitting, the stronger the effects of each hour per day of sitting time on several biomarkers (HDL cholesterol, triglycerides and PLG). This is consistent with the previous research that has aimed to examine or compare short and long sedentary bouts [19,21], but has been limited by the reliance on low-quality measures and other analytic issues, including the somewhat arbitrary divisions between short and long bouts. Though generally supportive that being sedentary for longer periods at a time may magnify the health risks of sedentary time, more research is needed to develop specific recommendations, such as how long is too long to be sitting without taking a break.

Measurement quality of the exposure variables was a key study strength. The exposure variables were measured over a requested 7-days, which is sufficient to produce reliable measures of total sedentary time over a 3-year period [48]. That said, to date, no studies have assessed the degree to which measures from a 7-day sedentary accumulation pattern measurement protocol reflect longer-term patterns of behavior and our results should be interpreted in consideration of this potential limitation. Future studies should consider longer measurement protocols. Key limitations were the cross-sectional design, which makes results subject to reverse-causality bias (e.g., cardiovascular disease, diabetes, and/or BMI could cause prolonged patterns of sitting accumulation), and the sample size, which was not chosen a priori and sometimes provided insufficient precision as indicated by the 95% confidence intervals of some associations that were not statistically significant containing effects of a magnitude ≥ MDIs. The sample, though covering a broad cross-section of Australian adults, was not population representative, with loss to follow-up prior to this third wave of data collection, and some biases in the subsampling and subsample participation, [34] with potential consequences both to internal and external validity. Residual confounding may exist from unmeasured variables and variables measured with error (e.g., educational status was not current as at 2011/12). Many hypotheses were tested, and some results could be spurious. Of all the findings, the most caution is warranted concerning glucose—the literature has mixed findings and our results varied depending on the measure and statistical adjustment choices. In general, the internal consistency within this study in the direction of the associations and the similarity between our findings and those of other studies suggest that most of our findings are sound.

This study adds important, robust evidence to a growing body of research supporting that in addition to high volumes of time spent sitting, the manner in which sitting time is accumulated has relevance for key areas of cardiovascular and metabolic health—lipid metabolism, glucose metabolism, and adiposity. Evidence concerning causation for long-term effects, such as from longitudinal and/or long-term intervention studies, is needed.

## Supporting information

S1 Fig Distributions of sitting bouts shown for one participant over a seven-day measurement period. (DOCX)

S1 Table List of all variables considered or adjusted for as potential confounders in multivariable models. (DOCX)

S2 Table Range of values within each quintile of sitting, sitting in 30+ min bouts, sit-stand transitions, usual bout duration, and alpha. (DOCX)

S3 Table Sociodemographic, behavioral, medical, cardiometabolic, and sitting-related characteristics of the final analytic sample, Australia, 2011/12. (DOCX)

S4 Table Statistical significance of interactions by age categories and gender in associations of sitting and prolonged sitting time, and sitting accumulation with cardio-metabolic biomarkers, adjusted for potential confounders (listed in S1 Table). (DOCX)

S5 Table Mean differences between Quartile 1 and Quartiles 2–5 for measures of adiposity and lipids by quintiles of sitting, prolonged sitting, sit-stand transitions, usual bout duration, and alpha after additional adjustment for MVPA; AusDiab (2011–12), n = 678. (DOCX)

S6 Table Mean differences between Quartile 1 and Quartiles 2–5 for blood pressure and measures of glucose control by quintiles of sitting, prolonged sitting, sit-stand transitions, usual bout duration, and alpha after additional adjustment for MVPA; AusDiab (2011–12), n = 678. (DOCX)

S7 Table Results from multivariable linear regression models testing effect modification of usual bout duration on associations of daily sitting time and cardiometabolic risk biomarkers, AusDiab (2011–12), n = 678. (DOCX)

## References

[1] Lozano R, Naghavi M, Foreman K, Lim S, Shibuya K, Aboyans V, et al Global and regional mortality from 235 causes of death for 20 age groups in 1990 and 2010: A systematic analysis for the Global Burden of Disease Study 2010. Lancet. 2012;380: 2095–2128. doi: 10.1016/S0140-6736(12)61728-0 23245604 10.1016/S0140-6736(12)61728-0 PMC10790329

[2] Sedentary Behavior Research Network. Letter to the Editor: Standardized use of the terms “sedentary” and “sedentary behaviours”. Appl Physiol Nutr Metab. 2012;37: 540–542. doi: 10.1139/h2012-024 22540258 10.1139/h2012-024

[3] Wilmot EG, Edwardson CL, Achana FA, Davies MJ, Gorely T, Gray LJ, et al Sedentary time in adults and the association with diabetes, cardiovascular disease and death: systematic review and meta-analysis. Diabetologia. Department of Cardiovascular Sciences, University of Leicester, Leicester Diabetes Centre, Leicester General Hospital, Gwendolen Road, Leicester, LE5 4PW, UK.; 2012;55: 2895–2905. doi: 10.1007/s00125-012-2677-z 22890825 10.1007/s00125-012-2677-z

[4] Thorp A a., Owen N, Neuhaus M, Dunstan DW. Sedentary behaviors and subsequent health outcomes in adults: A systematic review of longitudinal studies, 1996–2011. Am J Prev Med. Elsevier Inc.; 2011;41: 207–215. doi: 10.1016/j.amepre.2011.05.004 21767729 10.1016/j.amepre.2011.05.004

[5] Biswas A, Oh PI, Faulkner GE, Bajaj RR, Silver M a., Mitchell MS, et al Sedentary time and its association with risk for disease incidence, mortality, and hospitalization in adults. Ann Intern Med. 2015;162: 123–132. doi: 10.7326/M14-1651 25599350 10.7326/M14-1651

[6] Brocklebank LA, Falconer CL, Page AS, Perry R, Cooper AR. Accelerometer-measured sedentary time and cardiometabolic biomarkers: A systematic review. Prev Med (Baltim). Elsevier B.V.; 2015;76: 92–102. doi: 10.1016/j.ypmed.2015.04.013 25913420 10.1016/j.ypmed.2015.04.013

[7] Healy GN, Matthews CE, Dunstan DW, Winkler EAH, Owen N. Sedentary time and cardio-metabolic biomarkers in US adults: NHANES 2003–06. Eur Heart J. 2011;32: 590–7. doi: 10.1093/eurheartj/ehq451 21224291 10.1093/eurheartj/ehq451 PMC3634159

[8] The Australian Government. Australia’s Physical Activity and Sedentary Behaviour Guidelines. In: The Department of Health website. 2014.

[9] Chief Medical Officers of England, Scotland W and N. Start active, stay active. A report on physical activity for health from the four home countries’ Chief Medical Officers. London; 2011.

[10] Dunstan DW, Howard BJ, Bergouignan A, Kingwell BA, Owen N. Physiological implications of sedentary behavior: emerging human experimental evidence on reducing and breaking up sitting time In: Zhu W, Owen N, editors. Sedentary Behavior and Health: Concepts, Assessment & Intervention. Human Kinetics; 2016.

[11] Larsen RN, Kingwell B a., Sethi P, Cerin E, Owen N, Dunstan DW. Breaking up prolonged sitting reduces resting blood pressure in overweight/obese adults. Nutr Metab Cardiovasc Dis. Elsevier Ltd; 2014;24: 976–982. doi: 10.1016/j.numecd.2014.04.011 24875670 10.1016/j.numecd.2014.04.011

[12] Dempsey P, Sacre J, Owen N, Straznicky N, Cohen N, Kingwell B, et al Interrupting prolonged sitting reduces resting blood pressure in adults with type 2 diabetes. Hear Lung Circ. Australasian Society of Cardiac and Thoracic Surgeons and The Cardiac Society of Australia and New Zealand; 2015;24: S127–S128. doi: 10.1016/j.hlc.2015.06.033

[13] Dunstan DW, Kingwell B a, Larsen R, Healy GN, Cerin E, Hamilton MT, et al Breaking up prolonged sitting reduces postprandial glucose and insulin responses. Diabetes Care. 2012;35: 976–83. doi: 10.2337/dc11-1931 22374636 10.2337/dc11-1931 PMC3329818

[14] Peddie MC, Bone JL, Rehrer NJ, Skeaff CM, Gray AR, Perry TL. Breaking prolonged sitting reduces postprandial glycemia in healthy, normal-weight adults: a randomized crossover trial. Am J Clin Nutr. 2013;98: 358–66. doi: 10.3945/ajcn.112.051763 23803893 10.3945/ajcn.112.051763

[15] Henson J, Davies MJ, Bodicoat DH, Edwardson CL, Gill JMR, Stensel DJ, et al Breaking up prolonged sitting with standing or walking attenuates the postprandial metabolic response in post-menopausal women: A randomised acute study. Diabetes Care. 2015; In Press. doi: 10.2337/dc15-1240 26628415 10.2337/dc15-1240

[16] Dempsey PC, Owen N, Yates TE, Kingwell BA, Dunstan DW. Sitting Less and Moving More: Improved Glycaemic Control for Type 2 Diabetes Prevention and Management. Curr Diab Rep. Current Diabetes Reports; 2016;16: 114 doi: 10.1007/s11892-016-0797-4 27699700 10.1007/s11892-016-0797-4

[17] Chastin SFM, Egerton T, Leask C, Stamatakis E. Meta-analysis of the relationship between breaks in sedentary behavior and cardiometabolic health. Obesity. 2015;23: 1800–1810. doi: 10.1002/oby.21180 26308477 10.1002/oby.21180

[18] Dempsey PC, Blankenship JM, Larsen RN, Sacre JW, Sethi P, Straznicky NE, et al Interrupting prolonged sitting in type 2 diabetes: nocturnal persistence of improved glycaemic control. Diabetologia. Diabetologia; 2016; doi: 10.1007/s00125-016-4169-z 27942799 10.1007/s00125-016-4169-z

[19] Healy GN, Winkler E a. H, Brakenridge CL, Reeves MM, Eakin EG. Accelerometer-Derived Sedentary and Physical Activity Time in Overweight/Obese Adults with Type 2 Diabetes: Cross-Sectional Associations with Cardiometabolic Biomarkers. PLoS One. 2015;10: e0119140 doi: 10.1371/journal.pone.0119140 25775249 10.1371/journal.pone.0119140 PMC4361561

[20] Kim Y, Welk GJ, Braun SI, Kang M. Extracting Objective Estimates of Sedentary Behavior from Accelerometer Data: Measurement Considerations for Surveillance and Research Applications. PLoS One. 2015;10: e0118078 doi: 10.1371/journal.pone.0118078 25658473 10.1371/journal.pone.0118078 PMC4319840

[21] Falconer CL, Page AS, Andrews RC, Cooper AR. The Potential Impact of Displacing Sedentary Time in Adults with Type 2 Diabetes. Med Sci Sport Exerc. 2015; 1 doi: 10.1249/MSS.0000000000000651 26378943 10.1249/MSS.0000000000000651 PMC5131683

[22] Carson V, Wong SL, Winkler E, Healy GN, Colley RC, Tremblay MS. Patterns of sedentary time and cardiometabolic risk among Canadian adults. Prev Med (Baltim). Elsevier Inc.; 2014;65: 23–27. doi: 10.1016/j.ypmed.2014.04.005 24732719 10.1016/j.ypmed.2014.04.005

[23] Young DR, Hivert M-F, Alhassan S, Camhi SM, Ferguson JF, Katzmarzyk PT, Lewis CE, Owen N, Perry CK, Siddique J YC on behalf of the PAC of the C on L and CHC on CCC on E and PC on FG and TB and S. Sedentary behavior and cardiovascular morbidity and mortality: A science advisory from the American Heart Association. Circulation. 2016;134 doi: 10.1161/CIR.0000000000000440 27528691 10.1161/CIR.0000000000000440

[24] Healy G, Dunstan DW, Salmon J, Cerin E, Shaw J, Zimmet P, et al Breaks in sedentart time: beneficial associations with metabolic risk. Diabetes Care. 2008;31: 661–666. doi: 10.2337/dc07-2046 18252901 10.2337/dc07-2046

[25] Cooper AR, Sebire S, Montgomery A.A., Peters TJ, Sharp DJ, Jackson N, et al Sedentary time, breaks in sedentary time and metabolic variables in people with newly diagnosed type 2 diabetes. Diabetologia. 2012;55: 589–599. doi: 10.1007/s00125-011-2408-x 22167127 10.1007/s00125-011-2408-x

[26] Henson J, Yates T, Biddle SJH, Edwardson CL, Khunti K, Wilmot EG, et al Associations of objectively measured sedentary behaviour and physical activity with markers of cardiometabolic health. Diabetologia. 2013;56: 1012–1020. doi: 10.1007/s00125-013-2845-9 23456209 10.1007/s00125-013-2845-9

[27] Lyden K, Kozey Keadle SL, Staudenmayer JW, Freedson PS. Validity of two wearable monitors to estimate breaks from sedentary time. Med Sci Sports Exerc. Department of Kinesiology, University of Massachusetts, Amherst1 Department of Math and Statistics, University of Massachusetts, Amherst2.; 2012;44: 2243–2252. doi: 10.1249/MSS.0b013e318260c477 22648343 10.1249/MSS.0b013e318260c477 PMC3475768

[28] Barreira T V., Zderic TW, Schuna JM, Hamilton MT, Tudor-Locke C. Free-living activity counts-derived breaks in sedentary time: Are they real transitions from sitting to standing? Gait Posture. Elsevier B.V.; 2015;42: 70–72. doi: 10.1016/j.gaitpost.2015.04.008 25953504 10.1016/j.gaitpost.2015.04.008

[29] Kozey-Keadle S, Libertine A, Lyden K, Staudenmayer J, Freedson PS. Validation of wearable monitors for assessing sedentary behavior. Med Sci Sports Exerc. Department of Kinesiology, University of Massachusetts, Amherst, MA, USA.; 2011;43: 1561–1567. doi: 10.1249/MSS.0b013e31820ce174 21233777 10.1249/MSS.0b013e31820ce174

[30] Grant PM, Ryan CG, Tigbe WW, Granat MH. The validation of a novel activity monitor in the measurement of posture and motion during everyday activities. Br J Sports Med. Glasgow Caledonian University, School of Health and Social Care, Glasgow, UK. m.grant@gcal.ac.uk; 2006;40: 992–997. doi: 10.1136/bjsm.2006.030262 16980531 10.1136/bjsm.2006.030262 PMC2577473

[31] Dunstan DW, Zimmet PZ, Welborn T a, Cameron AJ, Shaw J, de Courten M, et al The Australian Diabetes, Obesity and Lifestyle Study (AusDiab)—methods and response rates. Diabetes Res Clin Pract. 2002;57: 119–129. doi: 10.1016/S0168-8227(02)00025-6 12062857 10.1016/s0168-8227(02)00025-6

[32] Barr E, Magliano D, Zimmet P, Polkinghorne K, Atkins R, Dunstan D, et al AUSDIAB 2005: The Australian Diabetes, Obesity and Lifestyle Study [Internet]. 2005 https://www.bakeridi.edu.au/Assets/Files/AUSDIAB_Report_2005.pdf

[33] Tanamas SK, Magliano DJ, Lynch B, Sethi P, Willenberg L, Polkinghorne KR, et al AUSDIAB 2012: The Australian Diabetes, Obesity and Lifestyle Study. 2013; 1–92. http://www.bakeridi.edu.au/AusDiabFindings

[34] Healy GN, Winkler EA, Owen N, Anuradha S, Dunstan DW. Replacing sitting time with standing or stepping: associations with cardio-metabolic risk biomarkers. Eur Heart J. 2015; EHV308 doi: 10.1093/eurheartj/ehv308 26228867 10.1093/eurheartj/ehv308

[35] Friedewald WT, Levy RI, Fredrickson DS. Estimation of the concentration of low-density lipoprotein cholesterol in plasma, without use of the preparative ultracentrifuge. Clin Chem. 1972;18: 499–502. 4337382

[36] Giles G, Ireland P. Dietary questionnaire for epidemiological studies (Version 2). 1996.

[37] Chastin SF, Winkler EA, Eakin EG, Gardiner PA, Dunstan DW, Owen N, et al Sensitivity to change of objectively-derived measures of sedentary behavior. Meas Phys Educ Exerc Sci. 2015;19: 138–147. doi: 10.1080/1091367X.2015.1050592

[38] Chastin SFM, Granat MH. Methods for objective measure, quantification and analysis of sedentary behaviour and inactivity. Gait Posture. 2010;31: 82–6. doi: 10.1016/j.gaitpost.2009.09.002 19854651 10.1016/j.gaitpost.2009.09.002

[39] Grant PM, Ryan CG, Tigbe WW, Granat MH. The validation of a novel activity monitor in the measurement of posture and motion during everyday activities. Br J Sports Med. 2006;40: 992–997. doi: 10.1136/bjsm.2006.030262 16980531 10.1136/bjsm.2006.030262 PMC2577473

[40] Willett W, Stampfer MJ. Total energy intake: implications for epidemiologic analyses. Am J Epidemiol. 1986;124: 17–27. 3521261 10.1093/oxfordjournals.aje.a114366

[41] Qi Q, Strizich G, Merchant G, Sotres-Alvarez D, Buelna C, Castañeda SF, et al Objectively Measured Sedentary Time and Cardiometabolic Biomarkers in US Hispanic/Latino Adults: The Hispanic Community Health Study/Study of Latinos (HCHS/SOL). Circulation. 2015;132: 1560–1569. doi: 10.1161/CIRCULATIONAHA.115.016938 26416808 10.1161/CIRCULATIONAHA.115.016938 PMC4618246

[42] Chastin SF, Winkler EA, Eakin EG, Gardiner PA, Dunstan DW, Owen N, et al Sensitivity to change of objectively-derived measures of sedentary behavior: supplementary material. In: Measurement in Physical Education and Exercise Science [Internet]. 2015 [cited 26 Jan 2016]. http://www.tandfonline.com/doi/suppl/10.1080/1091367X.2015.1050592/suppl_file/hmpe_a_1050592_sm0678.docx

[43] Page A, Peeters G, Merom D. Adjustment for physical activity in studies of sedentary behaviour. Emerg Themes Epidemiol. BioMed Central; 2015;12: 10 doi: 10.1186/s12982-015-0032-9 26161129 10.1186/s12982-015-0032-9 PMC4496859

[44] Schisterman EF, Cole SR, Platt RW. Overadjustment bias and unnecessary adjustment in epidemiologic studies. Epidemiology. 2009;20: 488–495. doi: 10.1097/EDE.0b013e3181a819a1 19525685 10.1097/EDE.0b013e3181a819a1 PMC2744485

[45] van der Berg JD, Stehouwer CDA, Bosma H, van der Velde JHPM, Willems PJB, Savelberg HHCM, et al Associations of total amount and patterns of sedentary behaviour with type 2 diabetes and the metabolic syndrome: The Maastricht Study. Diabetologia. 2016; doi: 10.1007/s00125-015-3861-8 26831300 10.1007/s00125-015-3861-8 PMC4779127

[46] Scheers T, Philippaerts R, Lefevre J. SenseWear-determined physical activity and sedentary behavior and metabolic syndrome. Med Sci Sports Exerc. 2013;45: 481–489. doi: 10.1249/MSS.0b013e31827563ba 23034646 10.1249/MSS.0b013e31827563ba

[47] Chastin SFM, Palarea-Albaladejo J, Dontje ML, Skelton DA. Combined effects of time spent in physical activity, sedentary behaviors and sleep on obesity and cardio-metabolic health markers: A novel compositional data analysis approach. PLoS One. 2015;10 doi: 10.1371/journal.pone.0139984 26461112 10.1371/journal.pone.0139984 PMC4604082

[48] Keadle SK, Shiroma EJ, Kamada M, Matthews CE, Harris TB, Lee I-M. Reproducibility of Accelerometer-Assessed Physical Activity and Sedentary Time. Am J Prev Med. Elsevier Inc.; 2017;52: 541–548. doi: 10.1016/j.amepre.2016.11.010 28062274 10.1016/j.amepre.2016.11.010 PMC5362292

