# Supplementary material for: Associations of sitting accumulation patterns with cardio-metabolic risk biomarkers in Australian adults
Source: PLoS One. 2017 Jun 29;12(6):e0180119. doi: 10.1371/journal.pone.0180119 (PMC5491133; doi:10.1371/journal.pone.0180119)
Supplement: S1 Fig — (DOCX) [file pone.0180119.s001.docx]

**S1 Fig: Distributions of sitting bouts shown for one participant over a seven-day measurement period.**


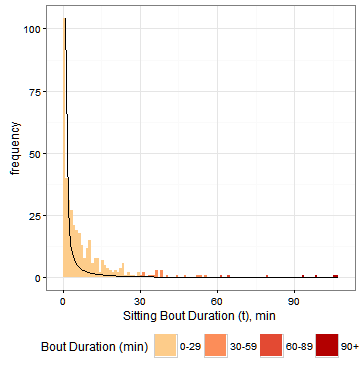

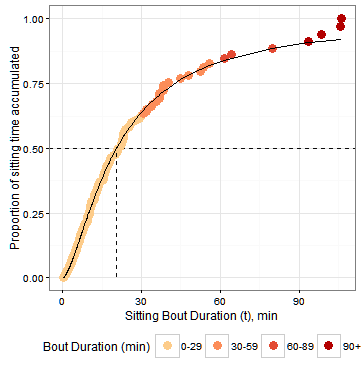

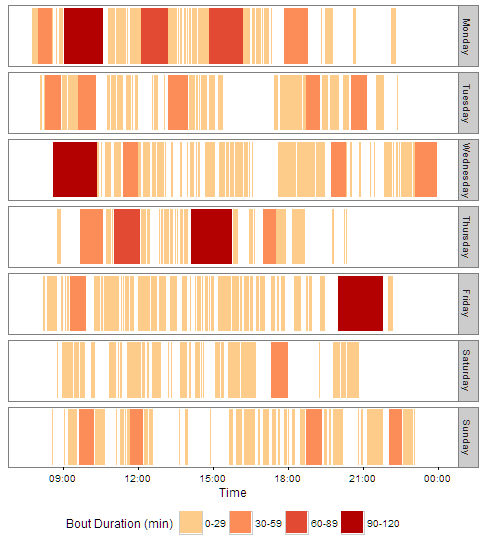


Examples of “breaks”

a) Frequency distribution of sitting bout duration

b) Cumulative distribution of sitting bout duration

c) Temporal distribution of sitting bouts

- Fitted accumulation curve

Observed % accumulated

- Fitted distribution (power law),

$y=\mathrm{Ct}^{-\alpha}$

Shaded area = total volume of sitting

time (frequency x duration)

Usual sitting bout duration (50% accumulation)

1. A number of sitting bouts of varying durations add up to ***total volume of sitting time*** as illustrated on the frequency distribution (above). Total volume of ***time spent sitting in bouts ≥30 minutes*** is a portion of this total volume of sitting time.
2. The parameter ***alpha*** summarizes this distribution. The higher the value of alpha, the steeper the slope. Alpha was estimated by maximum likelihood methods as $\hat{\alpha}=1+n\left[ \sum_{i=1}^{n} ln\frac{t_{i}}{t_{min}} \right]^{-1}$, where n= number of sitting bouts, t = bout duration (min) and $t_{min}$= shortest bout recorded by the monitor.
3. The midpoint (50%) of the cumulative distribution (above) that accompanies the frequency distribution is what we have termed ***usual bout duration***, and has been referred to as w50 or x50. It was calculated by non-linear regression estimating the following sigmoidal curve function: $y= \frac{t^{n}}{t^{n}+ X_{50}^{n}}$, where t = sitting bout duration (min), n= a free parameter, x50 = *usual bout duration* (min), and y=the proportion of sitting time accumulated in bouts ≤ t.
4. ***Breaks in sitting time*** are the non-sitting periods between each sitting bout, shown above. The number of breaks is approximately equal to the number of sitting bouts, and so requires correction for volume of sitting time to reflect the interruption of a fixed amount of sitting time.
5. A number of aspects of sedentary patterns visible in the temporal patterning are not captured by these pattern measures, e.g., when sitting occurs (morning/evening, or relative to meals).

**References**: Chastin SFM, Granat MH. Methods for objective measure, quantification and analysis of sedentary behaviour and inactivity. Gait Posture. 2010;31: 82–6. doi:10.1016/j.gaitpost.2009.09.002

Chastin SFM, Winkler E A. H., Eakin EG, Gardiner P A., Dunstan DW, Owen N, et al. Sensitivity to change of objectively-derived measures of sedentary behavior. Meas Phys Educ Exerc Sci. 2015;19: 138–147. doi:10.1080/1091367X.2015.1050592
