# Supplementary material for: Associations of sitting accumulation patterns with cardio-metabolic risk biomarkers in Australian adults
Source: PLoS One. 2017 Jun 29;12(6):e0180119. doi: 10.1371/journal.pone.0180119 (PMC5491133; doi:10.1371/journal.pone.0180119)
Supplement: S1 Table — (DOCX) [file pone.0180119.s002.docx]

| **S1 Table. List of all variables considered or adjusted for as potential confounders in multivariable models.** | |
| --- | --- |
| Model | Potential covariates considered or adjusted in final analyses |
| Considered for all outcomes | *Age* (as continuous [years] or categorical [35-44 years; 45-54 years; 55-64 years; 65-74 years; ≥75 years]) |
|  | *Gender* (male/female) |
|  | *Menopausal status* (post-menopausal/going through menopause/pre-menopausal/not applicable [male]) |
|  | *Contraceptive pill use* (yes/no/not applicable [male]) |
|  | *Blood pressure tablets* (yes/no) |
|  | *Cholesterol tablets* (yes/no) |
|  | *Diabetes medication* (yes/no) |
|  | *Ethnicity* (Australian or New Zealand/Other English speaking/Other) |
|  | *Present occupation or previous if not working* (managers or professionals/technical & trade or community & personal service/clerical & administrative or sales/machinery operator & driver or laborer/never worked or unknown ) |
|  | *Annual household income before tax* (< $30k/$30 to < $60k/$60k to < 100k/≥ $100k/refused or don’t know or missing) |
|  | *Employment status* (full time/part time/retired/other not working/missing) |
|  | *Fiber intake* (g/day) |
|  | *Fat*, *%E* |
|  | *Saturated fat*, *%E* |
|  | *Alcohol intake* (g/day) |
|  | *Sodium intake* (mg/day) |
|  | *Potassium intake* (mg/day) |
|  | *Fruit and vegetable serves* (serves/day) |
| Body mass index (kg/m^2^) | age category, gender, blood pressure tablets, cholesterol tablets, depression score category, diabetes medications, fat intake, energy-adjusted fiber intake |
| Waist circumference (cm) | age category, gender, blood pressure tablets, cholesterol tablets, depression score category, diabetes medication, height, employment status, saturated fat intake, energy intake, alcohol intake, sodium intake |
| Systolic blood pressure (mmHg) | age, gender/menopausal status, blood pressure tablets, main lifetime occupation, diabetes medication |
| Diastolic blood pressure (mmHg) | age category, gender, blood pressure tablets, ethnicity, main lifetime occupation, family history of diabetes, ownership of current residence, height, saturated fat intake, energy intake, alcohol intake, sodium intake |
| Fasting glucose (mmol/L) | age, gender/menopausal status, cholesterol tablets, main lifetime occupation, diabetes medication, housing type, sodium intake, calcium intake |
| HbA_1c_ (%) | age, gender/ menopausal status, blood pressure tablets, cholesterol tablets, depression score category, main lifetime occupation, diabetes medication, housing type, height, employment status, alcohol intake, fruit and vegetable intake |
| Total cholesterol (mmol/L) | age category, gender/menopausal status, cholesterol tablets, ethnicity, diabetes medication, saturated fat intake, alcohol intake |
| HDL cholesterol (mmol/L) | age category, gender/menopausal status, cholesterol tablets, smoking, housing type, main lifetime occupation, history of CVD, alcohol intake |
| LDL cholesterol (mmol/L) | age category, gender/menopausal status, cholesterol tablets, ethnicity, saturated fat, energy-adjusted fiber intake |
| Triglycerides (mmol/L) | age category, gender/menopausal status, cholesterol tablets, smoking, depression score category, ownership of current residence, income category, fruit and vegetable intake |
| 2-hour post load glucose (mmol/L) | age, gender, ethnicity, smoking, married or defacto, family history of diabetes, housing type, height, calcium intake |
