# Supplementary material for: Associations of sitting accumulation patterns with cardio-metabolic risk biomarkers in Australian adults
Source: PLoS One. 2017 Jun 29;12(6):e0180119. doi: 10.1371/journal.pone.0180119 (PMC5491133; doi:10.1371/journal.pone.0180119)
Supplement: S2 Table — (DOCX) [file pone.0180119.s003.docx]

| **S2 Table. Range of values within each quintile of sitting, sitting in 30+ min bouts, sit-stand transitions, usual bout duration, and alpha.** | | | | | | | | | | | | | | | |
| --- | --- | --- | --- | --- | --- | --- | --- | --- | --- | --- | --- | --- | --- | --- | --- |
|  | Quintile 1 | | | Quintile 2 | | | Quintile 3 | | | Quintile 4 | | | Quintile 5 | | |
|  | n | Mean (SD) | Min, Max | n | Mean (SD) | Min, Max | n | Mean (SD) | Min, Max | n | Mean (SD) | Min, Max | n | Mean (SD) | Min, Max |
| Total sitting time^a^, *h/day* | 135 | 11.1 (0.6) | 10.3, 13.2 | 136 | 9.8 (0.3) | 9.3, 10.2 | 135 | 8.9 (0.2) | 8.4, 9.2 | 136 | 7.9 (0.3) | 7.3, 8.3 | 136 | 6.3 (0.9) | 3.1, 7.2 |
| Prolonged sitting time^a^, *h/day* | 135 | 6.5 (1.0) | 5.3, 9.7 | 136 | 4.8 (0.3) | 4.3, 5.2 | 135 | 3.9 (0.2) | 3.5, 4.2 | 136 | 3.1 (0.2) | 2.6, 3.4 | 136 | 2.0 (0.5) | 0.5, 2.5 |
| Sit-stand transitions^b^, *n/day* | 136 | 35.6 (4.5) | 19.8, 41.8 | 136 | 45.3 (1.8) | 41.9, 48.2 | 135 | 52.1 (2.3) | 48.3, 55.8 | 136 | 60.2 (2.7) | 55.9, 64.8 | 135 | 75.5 (10.6) | 64.9, 120.6 |
| Usual bout duration, *min* | 135 | 40.5 (7.9) | 32.8, 73.8 | 136 | 29.5 (1.6) | 27.0, 32.7 | 135 | 24.9 (1.2) | 22.9, 26.9 | 136 | 21.1 (1.1) | 19.2, 22.8 | 136 | 16.3 (2.1) | 9.6, 19.1 |
| Alpha | 136 | 1.29 (0.01) | 1.225, 1.304 | 136 | 1.32 (0.01) | 1.305, 1.324 | 135 | 1.33 (0.01) | 1.325,1.342 | 136 | 1.36 (0.01) | 1.343, 1.367 | 135 | 1.40 (0.03) | 1.368, 1.516 |
| Table reports mean (standard deviation). | | | | | | | | | | | | | | | |
| ^a^ Variables adjusted for device wear time using the residuals method. | | | | | | | | | | | | | | | |
| ^b^ Variable adjusted for daily sitting time using the residuals method. | | | | | | | | | | | | | | | |
|  | | | | | | | | | | | | | | | |
