# Supplementary material for: Associations of sitting accumulation patterns with cardio-metabolic risk biomarkers in Australian adults
Source: PLoS One. 2017 Jun 29;12(6):e0180119. doi: 10.1371/journal.pone.0180119 (PMC5491133; doi:10.1371/journal.pone.0180119)
Supplement: S3 Table — (DOCX) [file pone.0180119.s004.docx]

| **S3 Table. Sociodemographic, behavioral, medical, cardiometabolic, and sitting-related characteristics of the final analytic sample, Australia, 2011/12.** | |
| --- | --- |
|  | Final analytic sample (n=678) |
| **Socio-demographic** |  |
| Age, *years* | 57.8 (9.8) |
| Age category, *n(%)* |  |
| 35-44 years | 68 (10) |
| 45-54 years | 191 (28) |
| 55-64 years | 238 (35) |
| 65-74 years | 142 (21) |
| ≥75 years | 39 (6) |
| Men, *n(%)* | 297 (45) |
| Height, *cm* | 169.4 (8.9) |
| Ethnicity, *n(%)* |  |
| Australia/New Zealand | 554 (82) |
| Other English speaking | 75 (11) |
| Other non-English speaking | 49 (7) |
| Married/defacto, *n(%)* | 524 (78) |
| Employment status, *n(%)* |  |
| Full time | 254 (37) |
| Part time | 148 (22) |
| Retired | 200 (30) |
| Other/not working/missing | 76 (11) |
| Occupation (current or previous if retired or not currently working) *n(%)* |  |
| Managers/Professionals | 282 (42) |
| Technical & Trade/Community & Personal service | 85 (13) |
| Clerical & Administrative/Sales | 159 (23) |
| Machinery operator & driver/Laborer | 45 (6) |
| Never worked/Unknown | 107 (16) |
| Housing (house), *n(%)* | 619 (92) |
| Owns residence (yes), *n(%)* | 602 (89) |
| Annual household income before taxes, *n(%)* |  |
| < $30k | 92 (14) |
| $30k to <$60k | 161 (24) |
| $60k to <100k | 145 (22) |
| >= $100k | 241 (36) |
| refused/don’t know/missing | 39 (5) |
| **Behavioral** |  |
| Dietary intake |  |
| Fat, *%E* | 34.8 (5.2) |
| Saturated fat, *%E* | 13.8 (2.8) |
| Energy intake, *MJ/day* | 7621.9 (2937.4) |
| Fiber intake, *g/day* | 21.4 (8.8) |
| Total alcohol, *g/day* | 13.5 (17.2) |
| Sodium, *g/day* | 2296.7 (377.4) |
| Potassium, *g/day* | 2859.2 (926.2) |
| Calcium, *mg/day* | 904.7 (326.2) |
| Fruit and vegetables, s*erves/day* | 3.6 (1.5) |
| Smoking status, *n(%)*^c^ |  |
| Never smoker | 383 (56) |
| Ex-smoker | 247 (37) |
| Current smoker | 48 (7) |
| **Medical** |  |
| Family history of diabetes, *n(%)* | 191 (28) |
| Diabetes medications, *n(%)* | 32 (5) |
| Blood pressure tablets, *n(%)* | 69 (10) |
| Cholesterol tablets, *n(%)* | 131 (20) |
| Oral contraceptives, *n(%)* |  |
| Not applicable (male) | 297 (45) |
| No | 143 (22) |
| Yes | 221 (33) |
| Prior CVD diagnosis ^d^, *n(%)* | 41 (6) |
| Menopause, *n(%)* |  |
| Post-menopausal | 219 (32) |
| Going through menopause | 58 (8) |
| Pre-menopausal | 104 (15) |
| Not applicable (male) | 297 (45) |
| **Cardio-metabolic biomarkers** |  |
| Body Mass Index, *kg/m^2^* | 27.4 (4.9) |
| Waist circumference, *cm* | 93 (13.7) |
| Systolic blood pressure, *mmHg* | 126.3 (17.3) |
| Diastolic blood pressure, *mmHg* | 72.7 (10.5) |
| Fasting blood glucose, *mmol/L* | 5.3 (0.73) |
| HbA_1c_ (IFCC), *mmol/L* | 5.6 (0.35) |
| HDL cholesterol, *mmol/L* | 1.6 (0.41) |
| LDL cholesterol, *mmol/L* | 3 (0.82) |
| Triglycerides, *mmol/L* | 1.3 (0.66) |
| 2-hr postload plasma glucose, *mmol/*L | 5.6 (2.02) |
| **Sitting time and sitting accumulation** |  |
| Total sitting time^a^, *h/day* | 8.8 (1.7) |
| Prolonged sitting time ^a^, *h/day* | 4 (1.6) |
| Sit-stand transitions^b^, *n/day* | 54.1 (14.5) |
| Usual bout duration, *min* | 26.2 (8.9) |
| Alpha | 1.3 (0.039) |
| Moderate to vigorous physical activity^c^, h/day | 1.2 (0.4) |
| Table reports mean (standard deviation) or n(%) for categorical variables where means, standard deviations, and % are corrected for the complex sampling design using linearized variance estimation. | |
| ^a^ Variables adjusted for device wear time using the residuals method. | |
| ^b^ Variable adjusted for daily sitting time using the residuals method. | |
| ^c^ Measured via activPAL as "stepping" equivalent to ≥ 3 METs. | |
