# Supplementary material for: Associations of sitting accumulation patterns with cardio-metabolic risk biomarkers in Australian adults
Source: PLoS One. 2017 Jun 29;12(6):e0180119. doi: 10.1371/journal.pone.0180119 (PMC5491133; doi:10.1371/journal.pone.0180119)
Supplement: S4 Table — (DOCX) [file pone.0180119.s005.docx]

| **S4 Table. Statistical significance of interactions by age categories and gender in associations of sitting and prolonged sitting time, and sitting accumulation with cardio-metabolic biomarkers, adjusted for potential confounders (listed in S1 Table)** | | | | | | | | | | | |
| --- | --- | --- | --- | --- | --- | --- | --- | --- | --- | --- | --- |
|  | Age interactions (*p-value*) | | | | |  | Gender interactions (*p-value*) | | | | |
|  | Total sitting time, h/day | Prolonged sitting time, h/day | Sit-stand transitions, n/day | Usual bout duration, *min* | Alpha |  | Total sitting time, h/day | Prolonged sitting time, h/day | Sit-stand transitions, n/day | Usual bout duration, *min* | Alpha |
| Body Mass index (kg/m2), β | 0.932 | 0.781 | 0.459 | 0.408 | 0.284 |  | **0.039** | **0.015** | **0.024** | 0.054 | **0.002** |
| Waist circumference (cm), β | 0.713 | 0.852 | 0.515 | 0.482 | 0.692 |  | **0.022** | **0.008** | 0.149 | **0.038** | **0.013** |
| HDL Cholesterol (mmol/L), β | 0.518 | 0.906 | 0.917 | 0.162 | 0.657 |  | 0.728 | 0.956 | 0.361 | 0.557 | 0.921 |
| LDL Cholesterol (mmol/L), β | 0.062 | 0.623 | 0.953 | 0.871 | **0.049** |  | 0.208 | 0.233 | 0.757 | 0.505 | 0.876 |
| Triglycerides (mmol/L), RR | 0.528 | 0.935 | 0.707 | 0.874 | 0.695 |  | 0.405 | 0.271 | 0.988 | 0.232 | 0.728 |
| Systolic BP (mmHg), β | 0.061 | 0.156 | 0.753 | 0.818 | 0.184 |  | 0.075 | **0.042** | 0.264 | 0.053 | 0.230 |
| Diastolic BP (mmHg), β | 0.337 | 0.537 | 0.713 | 0.959 | 0.270 |  | 0.146 | 0.103 | 0.833 | 0.281 | 0.281 |
| HbA1c (mmol/mol), RR | 0.958 | 0.897 | 0.624 | 0.878 | 0.273 |  | 0.680 | 0.797 | 0.109 | 0.351 | 0.868 |
| Glucose (mmol/L), RR | 0.055 | 0.182 | 0.577 | 0.473 | 0.164 |  | 0.284 | 0.512 | 0.075 | 0.793 | 0.617 |
| 2-hour post-load glucose (mmol/L), RR | 0.226 | 0.721 | 0.469 | 0.157 | 0.853 |  | 0.187 | 0.272 | 0.936 | 0.616 | 0.428 |

**Bolded** values indicate p<0.05
