# Supplementary material for: Associations of sitting accumulation patterns with cardio-metabolic risk biomarkers in Australian adults
Source: PLoS One. 2017 Jun 29;12(6):e0180119. doi: 10.1371/journal.pone.0180119 (PMC5491133; doi:10.1371/journal.pone.0180119)
Supplement: S6 Table — (DOCX) [file pone.0180119.s007.docx]

| **S6 Table. Mean differences between Quartile 1 and Quartiles 2 - 5 for blood pressure and measures of glucose control by quintiles of sitting, prolonged sitting, sit-stand transitions, usual bout duration, and alpha after additional adjustment for MVPA; AusDiab (2011-12), n=678^a^.** | | | | | | | | | | | |
| --- | --- | --- | --- | --- | --- | --- | --- | --- | --- | --- | --- |
|  | Quantile 1^b^ | Quantile 2 | | Quantile 3 | | Quantile 4 | | Quantile 5 | |  |  |
|  |  | Mean diff.^c^ | 95% CI | Mean diff.^c^ | 95% CI | Mean diff.^c^ | 95% CI | Mean diff.^c^ | 95% CI | P-for-trend | P-ovarall |
| **Systolic BP (mmHg)** |  |  |  |  |  |  |  |  |  |  |  |
| Total sitting time^d,e^ | referent | 0.68 | (-2.54,3.90) | -1.64 | (-5.34,2.06) | 1.42 | (-2.38,5.21) | 1.03 | (-1.86,3.92) | 0.394 | 0.560 |
| Prolonged sitting time^d^ | referent | 2.83 | (-2.01,7.67) | 4.89 | (0.23,9.55) | 2.42 | (-1.84,6.69) | 3.27 | (-1.25,7.79) | 0.209 | 0.290 |
| Sit-stand transitions^f^ | referent | -2.16 | (-5.71,1.39) | -1.87 | (-5.91,2.18) | -1.46 | (-6.03,3.12) | -0.57 | (-5.26,4.12) | 0.955 | 0.725 |
| Usual bout duration | referent | 0.17 | (-4.32,4.66) | 3.42 | (-0.81,7.64) | 0.99 | (-4.00,5.97) | 1.99 | (-2.30,6.27) | 0.364 | 0.571 |
| Alpha | referent | 1.32 | (-2.56,5.20) | 0.29 | (-4.52,5.09) | -0.22 | (-5.77,5.32) | 0.34 | (-4.43,5.11) | 0.883 | 0.940 |
| **Diastolic BP (mmHg)** |  |  |  |  |  |  |  |  |  |  |  |
| Total sitting time^d,e^ | referent | 2.21 | (-0.06,4.49) | -0.97 | (-3.50,1.56) | 0.40 | (-2.17,2.98) | -0.42 | (-3.00,2.16) | 0.375 | 0.078 |
| Prolonged sitting time^d^ | referent | 2.86 | (0.70,5.02) | 2.45 | (-0.30,5.20) | 1.36 | (-1.29,4.01) | 2.14 | (-0.62,4.90) | 0.497 | 0.176 |
| Sit-stand transitions^f^ | referent | 0.65 | (-1.56,2.87) | -0.45 | (-2.95,2.05) | -0.48 | (-3.10,2.13) | 0.07 | (-2.76,2.90) | 0.763 | 0.836 |
| Usual bout duration | referent | 0.69 | (-1.38,2.75) | 1.25 | (-1.23,3.73) | 0.91 | (-1.75,3.58) | 1.71 | (-0.93,4.35) | 0.265 | 0.783 |
| Alpha | referent | -0.43 | (-2.50,1.65) | -1.45 | (-4.11,1.20) | -1.76 | (-4.81,1.29) | -1.81 | (-4.53,0.90) | 0.174 | 0.710 |
| **HbA1c (mmol/mol)** |  |  |  |  |  |  |  |  |  |  |  |
| Total sitting time^d,e^ | referent | 0.05 | (-0.06,0.16) | 0.00 | (-0.09,0.08) | -0.05 | (-0.14,0.05) | 0.00 | (-0.10,0.09) | 0.280 | 0.466 |
| Prolonged sitting time^d^ | referent | 0.04 | (-0.07,0.15) | 0.01 | (-0.10,0.13) | 0.01 | (-0.07,0.09) | 0.01 | (-0.08,0.10) | 0.868 | 0.916 |
| Sit-stand transitions^f^ | referent | -0.03 | (-0.12,0.06) | 0.04 | (-0.09,0.17) | 0.03 | (-0.05,0.12) | 0.01 | (-0.09,0.12) | 0.402 | 0.564 |
| Usual bout duration | referent | 0.07 | (-0.02,0.16) | 0.08 | (-0.03,0.19) | 0.06 | (-0.03,0.16) | 0.05 | (-0.02,0.12) | 0.233 | 0.513 |
| Alpha | referent | 0.01 | (-0.09,0.12) | -0.03 | (-0.15,0.08) | -0.03 | (-0.13,0.08) | -0.04 | (-0.14,0.06) | 0.304 | 0.834 |
| **Fasting glucose (mmol/L)** |  |  |  |  |  |  |  |  |  |  |  |
| Total sitting time^d,e^ | referent | 0.13 | (-0.06,0.32) | 0.00 | (-0.17,0.17) | -0.09 | (-0.27,0.10) | -0.10 | (-0.32,0.11) | 0.069 | 0.146 |
| Prolonged sitting time^d^ | referent | 0.06 | (-0.12,0.23) | 0.11 | (-0.10,0.33) | -0.01 | (-0.16,0.14) | -0.05 | (-0.29,0.20) | 0.500 | 0.492 |
| Sit-stand transitions^f^ | referent | -0.07 | (-0.26,0.12) | 0.03 | (-0.19,0.26) | 0.07 | (-0.15,0.29) | 0.05 | (-0.13,0.22) | 0.249 | 0.427 |
| Usual bout duration | referent | 0.06 | (-0.12,0.24) | 0.11 | (-0.11,0.32) | 0.14 | (-0.06,0.34) | -0.01 | (-0.20,0.19) | 0.783 | 0.480 |
| Alpha | referent | -0.05 | (-0.27,0.18) | -0.28 | (-0.44,-0.13) | -0.14 | (-0.29,0.02) | -0.19 | (-0.34,-0.03) | **0.002** | **<.001** |
| **2-hour post-load glucose (mmol/L)** |  |  |  |  |  |  |  |  |  |  |  |
| Total sitting time^d,e^ | referent | -0.15 | (-0.66,0.35) | -0.19 | (-0.70,0.32) | -0.17 | (-0.63,0.28) | -0.24 | (-0.60,0.12) | 0.190 | 0.743 |
| Prolonged sitting time^d^ | referent | -0.10 | (-0.42,0.21) | -0.19 | (-0.59,0.20) | -0.16 | (-0.53,0.21) | 0.12 | (-0.30,0.55) | 0.716 | 0.462 |
| Sit-stand transitions^f^ | referent | -0.14 | (-0.54,0.26) | 0.01 | (-0.30,0.32) | -0.26 | (-0.69,0.18) | 0.00 | (-0.43,0.43) | 0.813 | 0.576 |
| Usual bout duration | referent | -0.09 | (-0.35,0.18) | -0.11 | (-0.52,0.29) | 0.01 | (-0.33,0.36) | 0.26 | (-0.12,0.64) | 0.171 | 0.296 |
| Alpha | referent | -0.36 | (-0.77,0.05) | -0.14 | (-0.45,0.18) | -0.57 | (-1.08,-0.07) | -0.48 | (-0.83,-0.12) | **0.019** | 0.062 |
| ^a^ Models of 2-hour post-load glucose had n=639. | | | | | | | | | | |  |
| ^b^ Participants in quintile 1 have the highest total sitting time / prolonged sitting time / the most prolonged pattern of sitting time accumulation. Quintile cutpoints are in S2 Table. | | | | | | | | | | |  |
| ^c^ Difference in adjusted mean in contrast to quintile 1, adjusted for age and gender, covariates (see S1 Table), and MVPA measured using from linear regression model with linearized variance estimation accounting for state/testing centre strata/clusters. | | | | | | | | | | |  |
| ^d^ Variables adjusted for device wear time using the residuals method.  ^e^ Associations are similar to those previously reported in Healy et al. Eur Heart J. 2015, differing slightly due to small differences in inclusion criteria and differences in the functional form of total sitting time. | | | | | | | | | | |  |
| ^f^ Variable adjusted for total sitting time using the residuals method. | | | | | | | | | | |  |
| **Bolded** p-values indicate statistically significant relations at p< 0.05. | | | | | | | | | | |  |
