# Supplementary material for: Associations of sitting accumulation patterns with cardio-metabolic risk biomarkers in Australian adults
Source: PLoS One. 2017 Jun 29;12(6):e0180119. doi: 10.1371/journal.pone.0180119 (PMC5491133; doi:10.1371/journal.pone.0180119)
Supplement: S7 Table — (DOCX) [file pone.0180119.s008.docx]

| **S7 Table. Results from multivariable^a^ linear regression models testing effect modification of usual bout duration on associations of daily sitting time and cardiometabolic risk biomarkers, AusDiab (2011-12), n=678^b^.** | | | | | | | | | |
| --- | --- | --- | --- | --- | --- | --- | --- | --- | --- |
|  | Daily Sitting Time^c^ | | | Usual Bout Duration^c^ | | | Daily Sitting Time^c^ by Usual Bout Duration^c^ | | |
|  | β or RR | 95% CI | p-value | β or RR | 95% CI | p-value | β or RR | 95% CI | p-value |
| Body Mass index (kg/m2), RR | 1.01 | (1.00,1.02) | 0.068 | 1.02 | (1.00,1.04) | 0.079 | 1.00 | (0.99,1.01) | 0.583 |
| Waist circumference (cm), β | 0.66 | (-0.03,1.36) | 0.062 | 1.16 | (-0.08,2.40) | 0.065 | -0.16 | (-0.83,0.51) | 0.636 |
| HDL Cholesterol (mmol/L), β | -0.04 | (-0.06,-0.03) | **<0.001** | 0.02 | (-0.01,0.06) | 0.180 | -0.02 | (-0.03,-0.01) | **0.005** |
| LDL Cholesterol (mmol/L), β | 0.03 | (-0.01,0.07) | 0.206 | -0.05 | (-0.13,0.03) | 0.231 | 0.00 | (-0.03,0.03) | 0.876 |
| Triglycerides (mmol/L), RR | 1.07 | (1.05,1.09) | **<0.001** | 0.97 | (0.93,1.02) | 0.228 | 1.02 | (1.00,1.04) | **0.032** |
| Systolic BP (mmHg), β | -0.12 | (-0.85,0.62) | 0.750 | -0.30 | (-2.13,1.53) | 0.740 | -0.21 | (-0.75,0.34) | 0.445 |
| Diastolic BP (mmHg), β | 0.56 | (0.12,1.01) | **0.015** | -0.78 | (-1.76,0.19) | 0.110 | -0.04 | (-0.39,0.31) | 0.817 |
| HbA1c (mmol/mol), RR | 1.00 | (1.00,1.01) | 0.216 | 1.00 | (0.99,1.00) | 0.416 | 1.00 | (1.00,1.00) | 0.800 |
| Glucose (mmol/L), RR | 1.01 | (1.00,1.02) | **0.013** | 0.99 | (0.98,1.01) | 0.327 | 1.00 | (0.99,1.01) | 0.513 |
| 2-hour post-load glucose (mmol/L), RR | 1.02 | (1.01,1.04) | **<0.001** | 0.97 | (0.94,1.01) | 0.119 | 1.02 | (1.00,1.03) | **0.042** |
| β = unstandardized regression coefficient; RR = relative rate. | | | | | | | | |  |
| RRs were computed by exponentiating beta coefficients from models where outcome variables were log-transformed. | | | | | | | | |  |
| ^a^ Models controlled for age, gender, and other potential covariates listed in S1 Table. | | | | | | | | |  |
| ^b^ Models of 2-hour post-load glucose had n=639. | | | | | | | | |  |
| ^c^ Variables were first mean centered. |  |  |  |  |  |  |  |  |  |
| **Bolded** p-values indicate statistically significant relations p < 0.05. | | | |  |  |  |  |  |  |
